# Supplementary material for: DMRforPairs: identifying Differentially Methylated Regions between unique samples using array based methylation profiles
Source: BMC Bioinformatics. 2014 May 15;15:141. doi: 10.1186/1471-2105-15-141 (PMC4046028; doi:10.1186/1471-2105-15-141)
Supplement: Additional file 2 — DMRforPairs output for the comparison of A431-MCF7 and NA17018-NA17105. Please start from the HTML files in each folder. Available via the BMC Bioinformatics website. [file 1471-2105-15-141-S2.zip › 1394847754114233_MOESM2_ESM/A431_MCF7/figures/5438.pdf]

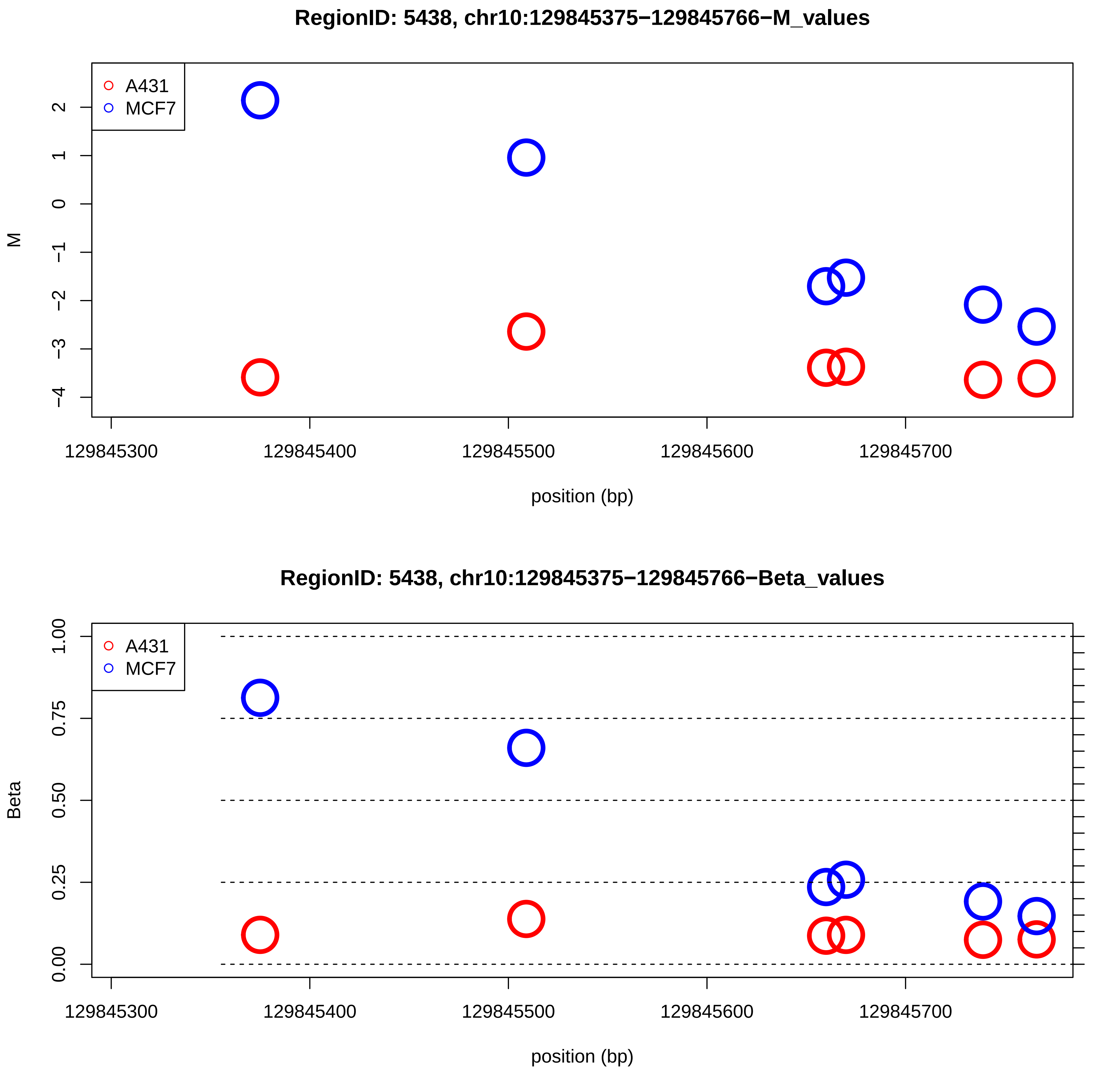

RegionID: 5438, chr10:129845375−129845766−M_values
A431
MCF7
M
2
1
0
−1
−2
−3
−4
129845300
129845400
129845500
129845600
129845700
position (bp)
RegionID: 5438, chr10:129845375−129845766−Beta_values
A431
MCF7
Beta
1.00
0.75
0.50
0.25
0.00
129845300
129845400
129845500
129845600
129845700
position (bp)

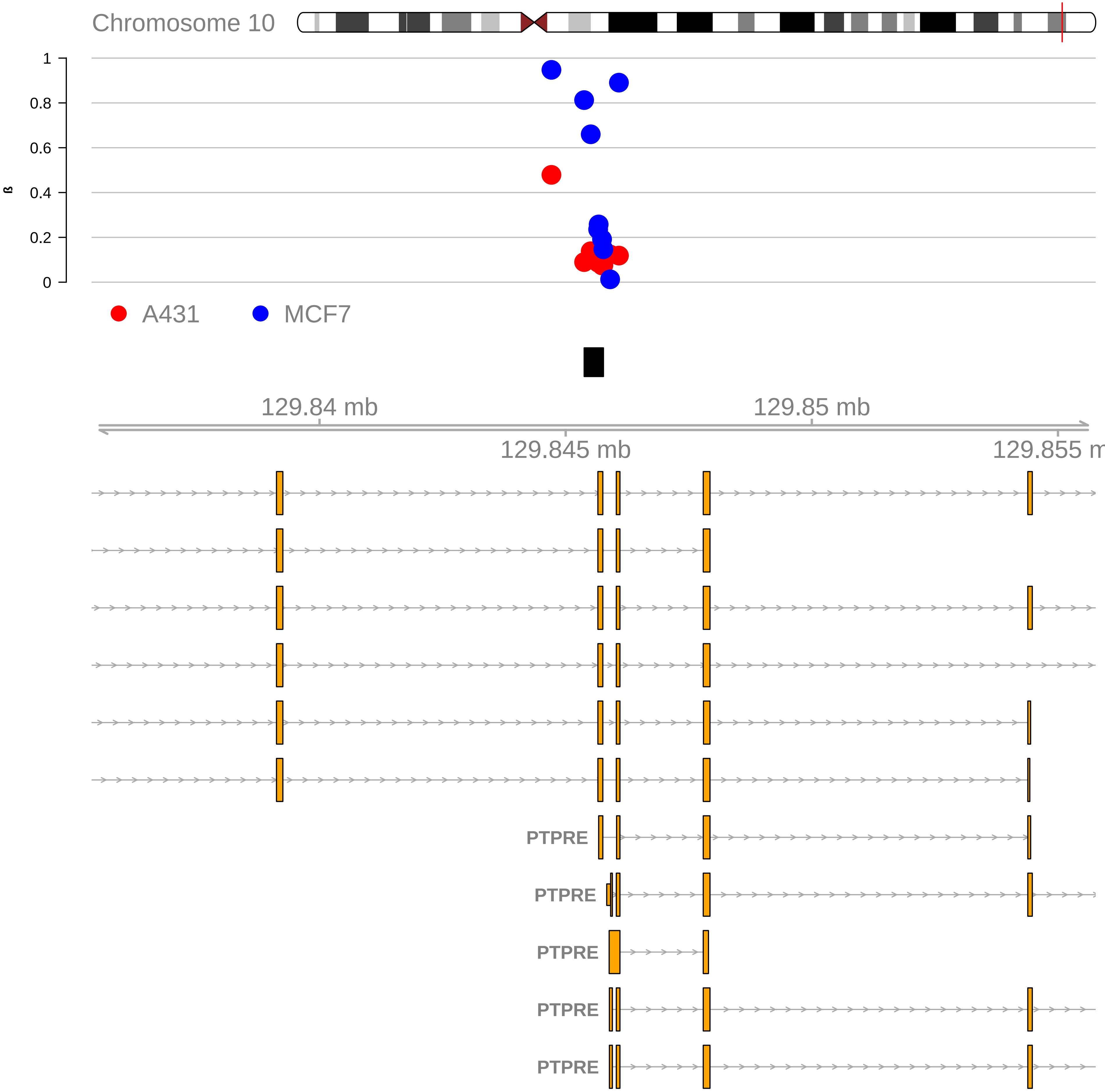

Chromosome 10
1
0.8
0.6
0.4
0.2
0
ß
A431
MCF7
129.84 mb
129.85 mb
129.845 mb
129.855 m
PTPRE
PTPRE
PTPRE
PTPRE
PTPRE
